# Introduction to Use of health impact metrics for programmatic decision making in global health

**DOI:** 10.1186/1471-2458-13-S2-S1

**Published:** 2013-06-17

**Authors:** Patricia H David

**Affiliations:** 1 Pathfinder International, 9 Galen Street, Suite 217, Watertown, MA 02472 USA

## 

Over the past twenty years, the international landscape for health programming in developing countries has changed dramatically, and global awareness of the consequences of poor health has grown. One result is the international commitment to the Millennium Development Goals (MDGs) that has put poverty reduction and health at the forefront of the global agenda. And, as new funding agencies and mechanisms (e.g., GFATM, GAVI, PEPFAR, PMI) appeared, attention focused more closely on the impact of their expenditures. Major donors - multilateral, bilateral and private - have embraced 'results-based programming'. They ask whether they are getting 'value for money'. And the commitment of national leaders to the MDGs confirms that they, as well as the donors, expect accountability for the significant expenditures now being made on health programs. Ultimately, what the global health community wants to know is which programs of service delivery can most efficiently improve health.

Without strong evaluations designed to measure impact, decision-makers lack sufficient evidence to fund expansion of service delivery or behavior change programs, and doing so without data on impact might even be considered unethical [1]. But there are many constraints to measuring the impact of large, complex programs, and studies to evaluate impact are expensive and take time [2].

The global health community has instead relied almost exclusively on measurements of intervention coverage, behaviors, and levels of mortality and fertility from household surveys to assess progress. This approach has a long tradition, starting in the 1970s with the World Fertility and Contraceptive Prevalence Surveys, and continues today with the Demographic and Health Surveys and the Multiple Indicator Cluster Surveys, which are the main vehicle for measuring progress toward many of the MDGs. But from the point of implementing organizations, surveys do not provide timely information, and rarely report below regional or provincial level, when more specific data are often needed for program management. Surveys are also expensive and they leave a large gap - a black box - between program inputs and their outcomes and impact. Many implementing organizations are looking for new approaches to express the impact, equity and breadth of their programs.

The papers in this Supplement provide a middle ground solution. They give us the opportunity to examine how two organizations that implement global health programs are addressing the need to assess performance, make strategic decisions about their programmatic priorities based on performance and goals, and at the same time be accountable to funders and national stakeholders. The papers provide details about how these program implementers are translating routinely-collected data on program outputs - either commodities distributed or services provided - into estimates of impact on health status, as expressed in disability-adjusted life years (DALYs) averted and on outcomes as expressed in changes in contraceptive prevalence and contribution to national indicators. The papers describe a range of organizational uses for data on impact, as well as analytic methods for examining equity in health status and program exposure that may be used to guide program development.

The supplement was stimulated by the growing interest in finding a set of common metrics that can be used at both the programmatic and global level for making decisions and setting priorities for action. As more organizations look to adopt such metrics, there is an increasing need for a published evidence base that can be used to inform and align measurement. After substantial investments in its own measurement program and at the request of other agencies wishing to adopt some of its core metrics, PSI worked with a group of partners and stakeholders to produce this supplement. The authors invite review of these metrics and their applications, hoping that the papers will engage the implementing community in debates on metrics that until now have largely been the purview of the academic community.

Thus, the supplement has several purposes. First, to share information on various models that can be used to translate outputs into impact estimates. Second, to provide some examples of how implementing organizations have applied these metrics for a variety of purposes. The papers included here identify some of the benefits of the current models as well as their limitations, and expose measurement gaps that remain. Finally, the papers identify some of the challenges for implementing organizations posed by the requirements of the models. The Commentaries that follow also highlight some of the challenges that face the global health community in our quest for data to inform program and policy decisions.

The first paper in the series, by Longfield and colleagues [3], describes the application of the DALYs averted metric to assess performance across PSI's global portfolio, describing its use to improve the organization's strategic decisions. This illustration is followed by a paper by Yang and colleagues [4] that provides the details of the DALYs averted models: a description of the structure, assumptions and model parameters and data inputs for two types of DALYs averted models. Two worked examples using PSI data are also provided, with an extended discussion of the challenges of customizing and updating the models, as well as limitations and plans for future improvements.

Next, the paper by Montagu and colleagues illustrates the utility of the DALYs averted metric for making comparisons among programs, as well as assessing program growth and impact using data from a large number of social franchise programs that deliver a variety of services and are managed by many different implementing organizations [5]. The paper makes the case that despite challenges in quality of data currently reported by some programs, DALYs averted are a promising opportunity to promote data sharing and transparency among the social franchise community.

The final papers by Weinberger and colleagues [6] and Chakraborty and colleagues [7] describe two other approaches. In the first, Weinberger describes how Marie Stopes International (MSI)'s Impact 2 model converts data on contraceptive service provision into estimates of numbers of family planning users, and, while allowing for factors such as population growth and a number of other adjustments, estimates the attributable contribution of the organization to national levels of contraceptive prevalence without recourse to surveys. In the final paper, Chakraborty and colleagues demonstrate how a combination of analytic methods for assessing equity in health behavior outcomes and intervention exposures - the concentration index and wealth quintiles - is useful for programmatic decision making. Employing characteristics of both methods, social marketers and other implementers can monitor the equity of their programs and improve targeting when the poor are not being reached.

For many organizations with limited resources, there is a tension between the need to be accountable to donors, to efficiently use funds for programmatic activities, and to learn what works best. The work described in the papers that follow required considerable investment in modeling expertise, as well as in collecting comparable data from multiple countries and projects. Many organizations that implement very disparate health programs on a smaller scale may find the data requirements for these models daunting. And moving from service outputs to health impact omits several layers of important information that is also needed for managing performance and learning from program results. The need for effectiveness evaluations, as well as for more complex impact evaluations, will not diminish.

The organizational uses of the methods and models described here for - program planning, for strategy development, for advocacy - and the opportunity to promote collaboration across organizations through use of standardized metrics are promising. The potential to gain a better understanding of whether health programs are well-positioned to meet their ultimate goal of improving health is definitely worth exploring.

## List of abbreviations

MDGs: Millennium Development Goals; GFATM: Global Fund to fight AIDS; Tuberculosis and Malaria; GAVI: Global Alliance for Vaccines and Immunization (GAVI); PEPFAR: President's Emergency Plan for AIDS Relief; PMI: President's Malaria Initiative; DALYs: disability-adjusted life years; PSI: Population Services International; MSI: Marie Stopes International

## Competing interests

The author declares that she has no competing interests.

## Declarations

This article is part of the supplement of *BMC Public Health *Volume 13, Supplement 2, 2013: Use of health impact metrics for programmatic decision making in global health. Population Services International, a registered non-profit organization, provided the funding for the publication of this supplement. The full contents of the supplement are available online at http://www.biomedcentral.com/bmcpublichealth/supplements/13/S2.

## References

[B1] Evaluation Gap Working Group When will we ever learn? Improving lives through impact evaluation 2006 Washington DC: Center for Global Development

[B2] Bryce J Victora CG MCE-IMCI Technical Advisors Ten methodological lessons from the Multi-Country Evaluation of Integrated Management of Childhood Illness Health Policy Plan 2005 20 Suppl 1 i94 i105 16306075 10.1093/heapol/czi056

[B3] Longfield K Smith B Gray R Ngamkitpaiboon L Vielot N Putting health metrics into practice: using the disability-adjusted life year for strategic decision making BMC Public Health 2013 13 Suppl 2 S2 23902655 10.1186/1471-2458-13-S2-S2 PMC3684549

[B4] Yang H Duvall S Ratcliffe A Jeffries D Stevens W Modeling health impact of global health programs implemented by Population Services International BMC Public Health 2013 13 Suppl 2 S3 23902668 10.1186/1471-2458-13-S2-S3 PMC3684543

[B5] Montagu D Ngamkitpaiboon L Duvall S Ratcliffe A Applying the disability-adjusted life year (DALY) to track health impact of social franchise programs in low- and middle-income countries BMC Public Health 2013 13 Suppl 2 S4 23902679 10.1186/1471-2458-13-S2-S4 PMC3684545

[B6] Weinberger MB Fry K Boler T Hopkins K Estimating the contribution of a service delivery organisation to the national modern contraceptive prevalence rate: Marie Stopes International's Impact 2 model BMC Public Health 2013 13 Suppl 2 S5 23902699 10.1186/1471-2458-13-S2-S5 PMC3684538

[B7] Charkraborty NM Firestone R Bellows N Equity monitoring for social marketing: use of wealth quintiles and the concentration index for decision making in HIV prevention, family planning, and malaria programs BMC Public Health 2013 13 Suppl 2 S6 10.1186/1471-2458-13-S2-S6 PMC3684531 23902715

